# Toll-like Receptor 7 (TLR7) Is Expressed in Adipocytes and the Pharmacological TLR7 Agonist Imiquimod and Adipocyte-Derived Cell-Free Nucleic Acids (cfDNA) Regulate Adipocyte Function

**DOI:** 10.3390/ijms23158475

**Published:** 2022-07-30

**Authors:** Miriam Thomalla, Andreas Schmid, Julia Hehner, Sebastian Koehler, Elena Neumann, Ulf Müller-Ladner, Andreas Schäffler, Thomas Karrasch

**Affiliations:** 1 Department of Internal Medicine III, Justus-Liebig-University Giessen, 35390 Gießen, Germany; miriam.thomalla@innere.med.uni-giessen.de (M.T.); andreas.schmid@innere.med.uni-giessen.de (A.S.); hehner@staff.uni-marburg.de (J.H.); sebastian.koehler@med.uni-giessen.de (S.K.); andreas.schaeffler@innere.med.uni-giessen.de (A.S.); 2 Department of Rheumatology and Clinical Immunology, Justus-Liebig-University Giessen, Campus Kerckhoff, 61231 Bad Nauheim, Germany; e.neumann@kerckhoff-klinik.de (E.N.); u.mueller-ladner@kerckhoff-klinik.de (U.M.-L.)

**Keywords:** TLR7, adipocyte, inflammation, adipose tissue, adipokine, pattern recognition receptor, cfDNA

## Abstract

Endosome-localized Toll-like receptors (TLRs) 3 and 9 are expressed and functionally active in adipocytes. The functionality and role of TLR7 in adipocyte biology and innate immunity of adipose tissue (AT) is poorly characterized. We analyzed TLR7 mRNA and protein expression in murine 3T3-L1 and primary adipocytes, in co-cultures of 3T3-L1 adipocytes with murine J774A.1 monocytes and in human AT. The effects of TLR7 agonists imiquimod (IMQ) and cell-free nucleic acids (cfDNA) on adipokine concentration in cell-culture supernatants and gene expression profile were investigated. We found that TLR7 expression is strongly induced during adipocyte differentiation. TLR7 gene expression in adipocytes and AT stroma-vascular cells (SVC) seems to be independent of TLR9. IMQ downregulates resistin concentration in adipocyte cell-culture supernatants and modulates gene expression of glucose transporter Glut4. Adipocyte-derived cfDNA reduces adiponectin and resistin in cell-culture supernatants and potentially inhibits Glut4 gene expression. The responsiveness of 3T3-L1 adipocytes to imiquimod is preserved in co-culture with J774A.1 monocytes. Obesity-related, adipocyte-derived cfDNA engages adipocytic pattern recognition receptors (PRRs), modulating AT immune and metabolic homeostasis during adipose inflammation.

## 1. Introduction

The innate immune system plays a crucial role in obesity-related metabolic inflammation (metaflammation), adipose inflammation and local and systemic insulin resistance [1,2,3]. Obesity is associated with the upregulated expression of multiple Toll-like receptors (TLRs) in murine models [4]. Recently, nucleic acid-sensing by various TLRs, in particular by TLR9, has been implicated in obesity and insulin resistance in both murine and human studies [5,6,7]. TLR9 belongs to a family of endosome-localized Toll-like receptors, with TLR7, TLR8 and TLR3 being the other family members [8]. Of note, the TLR7^−/−^-mice exhibit less metabolic inflammation and improved glucose tolerance in murine models of diet-induced obesity [9]. The TLR7 expression in adipose tissues is upregulated in human obesity, and is reduced after bariatric surgery [10]. Thus, TLR7 has been proposed to play a significant role in metabolically induced inflammation in obesity.

Physiologically, TLR7 recognizes mono-nucleosides (preferentially guanosine with synergistic activation by other mono-nucleosides, such as adenosine) together with uridine-rich (poly-U) single-stranded RNA (ssRNA) [11,12]. Originally interpreted to be a pattern-recognition receptor (PRR) sensing foreign nucleic acids, TLR7 has recently been proposed to recognize physiological ligands as well, in particular cell-free nucleic acids (cfDNA), which are increased in human obesity [9]. Of note, Nishimoto et al. demonstrated that cfDNA released from adipocytes due to obesity-related adipocyte degeneration promotes macrophage infiltration into adipose tissues via the TLR9 signaling, leading to adipose inflammation [6]. Thus, TLR7 and TLR9 potentially play interdependent roles in the inflammatory and metabolic homeostasis of adipose tissue compartments. However, the contribution of individual cell types within the adipose tissue compartments (e.g., the stroma-vascular cell fraction as opposed to the adipocytes themselves) remains a matter of debate. While we recently demonstrated a significant expression of functional TLR9 in adipocytes [13], only sparse data are available on the overall expression of TLR7 in adipocytes in obesity [10].

Our study therefore aimed to provide a detailed evaluation of TLR7 mRNA and protein expression in adipocytes. Furthermore, we aimed to analyze the effects of the pharmacological TLR7 agonist imiquimod, as well as the proposed ligand cfDNA, on adipocyte gene expression and adipokine concentration in cell-culture supernatants. Here we show that adipocyte differentiation induces the TLR7 mRNA and protein expression, which is increased in human visceral tissue as compared to the subcutaneous adipose tissue. The treatment of adipocytes with the pharmacological TLR7 agonist imiquimod, as well as the proposed ligand cell-free nucleic acids (cfDNA), reduces the concentration of the adipokines, resistin and adiponectin, in cell-culture supernatants, while modulating the Glut4 expression in adipocytes; adipocyte expression of TLR7 seems to be independent of the TLR9; and TLR7 expression in human adipose tissues negatively correlates to systemic resistin levels. These results add endosome-localized TLR7 as an additional candidate participating in the adipocyte-dependent innate-immune responses to inflammatory stimuli in the context of obesity, insulin resistance and metabolic inflammation.

## 2. Results

### 2.1. TLR7 Gene Expression Is Induced during 3T3-L1 Adipocyte Differentiation and TLR7 Protein Is Expressed in 3T3-L1 Adipocytes

**Figure 1:**
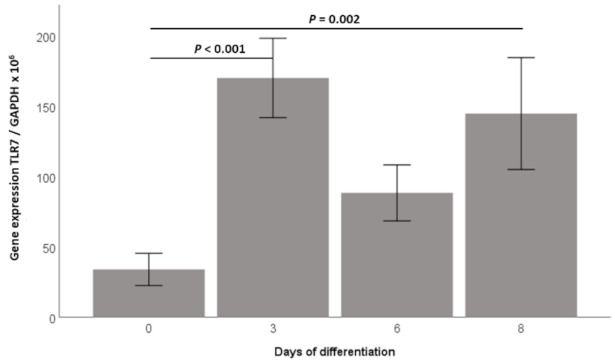
TLR7 gene expression is induced during 3T3-L1 adipocyte differentiation. TLR7 expression was investigated in 3T3-L1 fibroblasts (day 0) and pre-adipocytes (days 3 and 6) during differentiation into mature adipocytes (day 8). TLR7 mRNA levels (analyzed by RT-PCR) exhibited an early and significant increase and remained elevated throughout the adipocytic differentiation process (*n* = 11 each).

**Figure A1:**
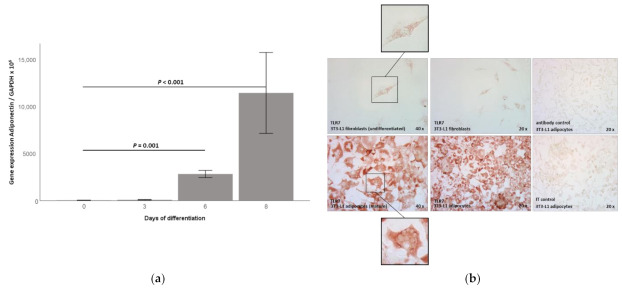
Adiponectin mRNA gene expression is induced during hormonally induced 3T3-L1 adipocyte differentiation, and TLR7 protein is expressed in 3T3-L1 adipocytes. Adiponectin expression was investigated in 3T3-L1 fibroblasts (day 0) and pre-adipocytes (days 3 and 6) during differentiation into mature adipocytes (day 8). (**a**) Adiponectin mRNA levels (analyzed by RT-PCR) exhibited an strong and significant increase, indicating successful adipocytic differentiation (*n* = 11 each); (**b**) Immunocytochemistry demonstrated a strong induction of TLR7 protein during adipocyte differentiation, which appeared to be predominantly in the cytoplasm of adipocytes (representative sections are shown, inserts show higher magnification of individual cells).

TLR7 has recently been suggested as participating in metabolically induced inflammation (“metaflammation”) in adipose tissue compartments [9]. Thus far, only sparse data are available on the expression and no data are available on the functionality of the TLR7 signaling in adipocytes. We therefore investigated the TLR7 mRNA expression during hormonally induced 3T3-L1 adipocyte differentiation in vitro. The TLR7 mRNA expression showed very low levels in preadipocytes (day 0), however, we found a strong induction of the TLR7 mRNA early at day 3 of adipocyte differentiation (*p* < 0.001, Figure 1), which lasted through day 8 of adipocyte differentiation (*p* = 0.002, Figure 1). Successful adipocyte differentiation was controlled by light-microscopy showing a spherical adipocyte phenotype with extensive lipid droplet accumulation; additionally, we found a strong induction of adiponectin mRNA proving successful adipocyte differentiation (Appendix A, Figure A1a). Similarly, the TLR7 protein was barely detectable by immunohistochemical analyses at day 0 of adipocyte differentiation, whereas there was a strong immunohistochemical signal revealing the adipocytic TLR7 protein expression at day 8 of differentiation (Appendix A, Figure A1b). The immunocytochemical signal appeared to be localized predominantly in the cytoplasm of adipocytes, matching its endosomal localization in other cell types (Appendix A, Figure A1b, inserts) [8].

### 2.2. TLR7 mRNA Is Expressed in the SVC and the Adipocyte Fraction of Intra-Abdominal Adipose Tissue (AT) in TLR9^wt/wt^ and in TLR9^−/−^ Mice

**Figure 2:**
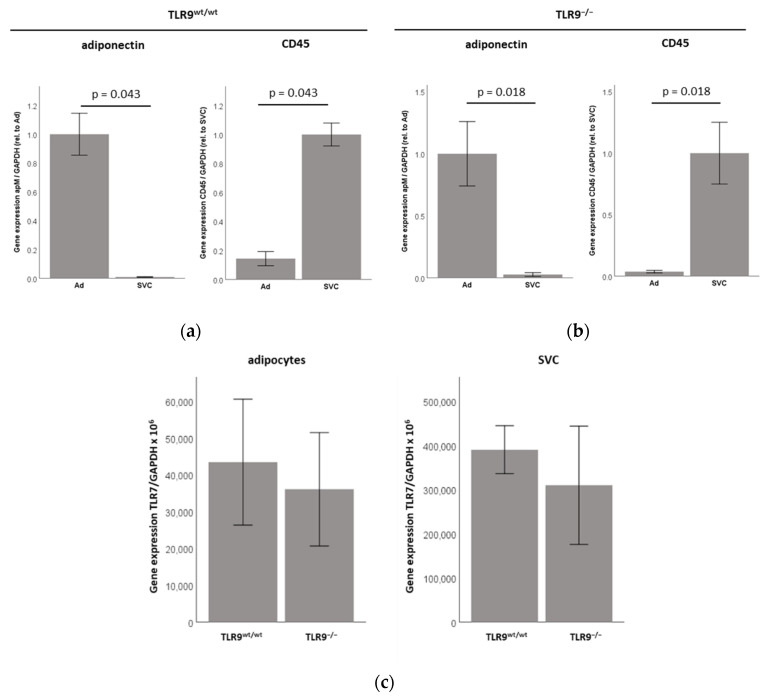
TLR7 mRNA is expressed in the stroma-vascular cell (SVC) and the adipocyte (Ad) fraction of intra-abdominal adipose tissue (AT) in TLR9^wt/wt^ and in TLR9^−/−^ mice. Adipocytes (Ad) and stroma-vascular cells (SVC) were isolated from intra-abdominal adipose tissue compartments of female TLR9^wt/wt^ and TLR9^−/−^ mice. (**a**,**b**) In preliminary experiments, the purity of adipocytes versus SVC after our isolation procedure was assessed by real-time PCR using adiponectin for the adipocyte fraction, and using CD45 (indicating leukocyte lineage) for the SVC fraction of cells in both genotypes: Adiponectin was almost exclusively expressed in the adipocyte fraction of cells, indicating that we reached a high purity of adipocytes during our isolation procedure. CD45, on the other hand, was almost exclusively expressed in the SVC fraction of cells, indicating that—as expected—this fraction contained a significant number of immune cells, which were absent in the adipocyte fraction of cells (*n* = 5–7); (**c**) TLR7 mRNA expression (relative to GAPDH expression levels) was significantly stronger in the SVC fraction as compared to the adipocyte (Ad) fraction, however, no significant difference was found in TLR7 mRNA expression levels in adipocytes or in SVCs between TLR9^wt/wt^ versus TLR9^−/−^ mice (*n* = 5–7).

Next, we investigated the TLR7 mRNA expression in the adipocytes, as compared to the stroma-vascular cell fraction of murine intra-abdominal adipose tissue (AT). Since we recently demonstrated that the adipocytes express the functional TLR9 [13], and since both TLR7 and TLR9 are intracellular toll-like receptors, and they have been suggested to play interdependent roles in adipose tissue physiology [9,13], we evaluated the TLR7 mRNA expression in the adipocytes and the SVC fraction within the intra-abdominal adipose tissues of both TLR9^wt/wt^ versus TLR9^−/−^ mice. After tissue resection, the cell fractions were prepared, as previously described [13]. In preliminary experiments, the purity of the adipocytes versus SVC after our isolation procedure was assessed by real-time PCR, using adiponectin for the adipocyte fraction, and using CD45 (indicating leukocyte lineage) for the SVC fraction of the cells in both of the genotypes: Adiponectin was almost exclusively expressed in the adipocyte fraction of the cells, indicating that we reached a high purity of adipocytes during our isolation procedure. The CD45, on the other hand, was almost exclusively expressed in the SVC fraction of the cells, indicating that—as expected—this fraction contained a significant number of immune cells, which were absent in the adipocyte fraction of the cells (Figure 2a,b).

**Figure A2:**
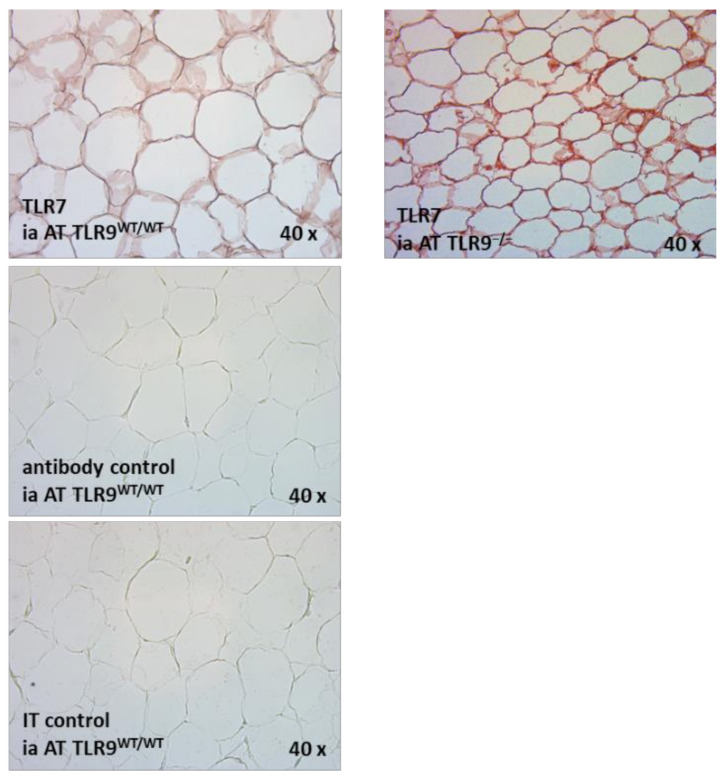
TLR7 protein is expressed in intra-abdominal adipose tissue (AT) in TLR9^wt/wt^ and in TLR9^−/−^ mice. TLR7 protein expression was detected and visualized via immunohistochemical methods in intra-abdominal (ia) adipose tissue from TLR9^wt/wt^ and TLR9^−/−^ mice (representative sections are shown).

As expected, the TLR7 mRNA expression (relative to GAPDH expression levels) was significantly stronger in the SVC fraction as compared to the adipocyte fraction, however, we also found significant TLR7 mRNA expression levels in the adipocytes themselves (Figure 2c). Of note, no significant difference was found in the TLR7 mRNA expression levels in adipocytes or in the SVCs between the TLR9^wt/wt^ versus TLR9^−/−^ mice (Figure 2c). Immunohistochemical studies demonstrated that the TLR7 protein is expressed in intra-abdominal adipose tissue of TLR9^wt/wt^ and TLR9^−/−^ mice (Appendix A, Figure A2). However, our immunohistochemical analyses did not allow a quantitative assessment or comparisons of the TLR7 protein expression levels between different groups of animals. These analyses should be the focus of future extensive studies. The representative immunohistochemical analyses of the TLR7 protein expression in the intra-abdominal adipose tissue of TLR9^wt/wt^ versus TLR9^−/−^ mice are shown in Appendix A, Figure A2.

### 2.3. TLR7 Agonist Imiquimod Inhibits Adiponectin and Resistin Accumulation in 3T3-L1 Adipocyte Cell-Culture Supernatants

**Figure 3:**
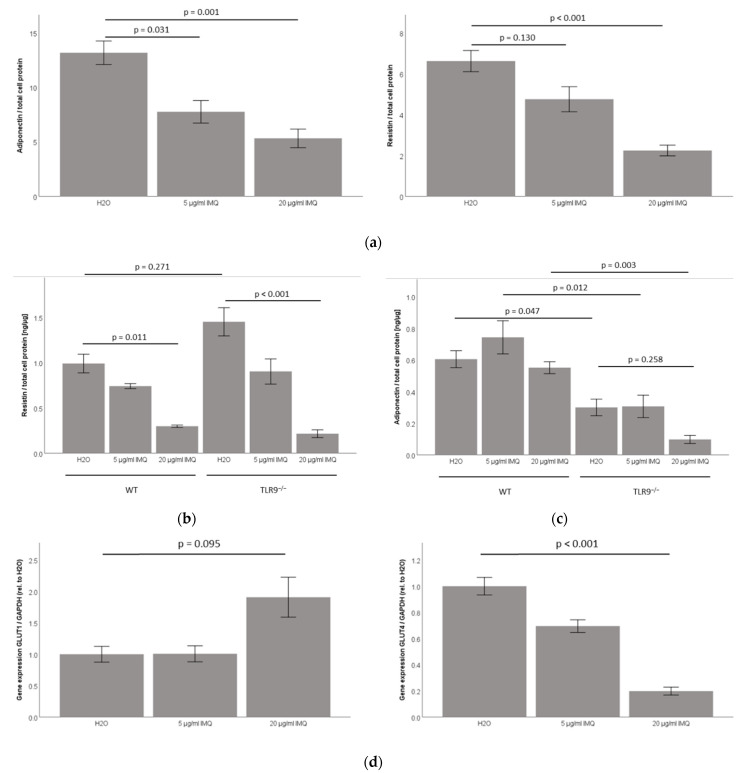
TLR7 agonist imiquimod inhibits adiponectin and resistin protein accumulation in 3T3-L1 adipocyte cell-culture supernatants, inhibits resistin accumulation in primary murine TLR9^wt/wt^ and TLR9^−/−^ subcutaneous adipocyte cell-culture supernatants and inhibits Glut4 mRNA expression in 3T3-L1 adipocytes in vitro. Mature 3T3-L1 adipocytes and primary adipocytes derived from subcutaneous adipose tissue of female TLR9^−/−^ and TLR9^wt/wt^ (WT) mice were treated with TLR7 agonist imiquimod (IMQ) for 18 h. (**a**) In 3T3-L1 adipocytes, supernatant adiponectin and resistin levels were downregulated significantly and in a dose-dependent manner by IMQ treatment (*n* = 6); (**b**) in primary cells from murine adipose tissues, resistin concentration in cell-culture supernatants was significantly downregulated by IMQ (*n* = 4–6) in both TLR9^−/−^ and TLR9^wt/wt^ adipocytes; (**c**) adiponectin concentration in cell-culture supernatants remained unaffected by IMQ treatment in both TLR9^−/−^ and TLR9^wt/wt^ adipocytes, while adiponectin levels were generally decreased in TLR9^−/−^ as compared to TLR9^wt/wt^ adipocytes (*n* = 4–6); (**d**) GLUT4 mRNA levels were strongly and dose-dependently decreased in IMQ-stimulated 3T3-L1 adipocytes, whereas GLUT1 levels were not significantly affected by IMQ treatment (*n* = 11).

**Figure A3:**
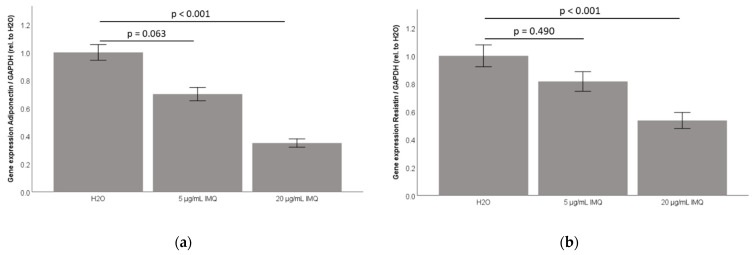
Imiquimod reduces both adiponectin and resistin mRNA levels in 3T3-L1 adipocytes in vitro. Mature 3T3-L1 adipocytes (*n* = 11–12 wells each) were treated with different doses of TLR7 agonist imiquimod (IMQ) for 18 h as described in the Materials and Methods section. After isolation of mRNA applying the RNeasy^®^ Mini Kit (Qiagen, Hilden, Germany), gene expression levels of murine adiponectin and resistin were quantified by reverse transcription and real-time PCR (RT-PCR) of the corresponding cDNA and normalized to GAPDH, as described in the Methods section. Imiquimod reduced both (**a**) adiponectin and (**b**) resistin mRNA levels.

Since the TLR7 mRNA and protein expression are induced during the differentiation in 3T3-L1 adipocytes (Figure 1; Appendix A, Figure A1b), we next investigated if the TLR7 signaling is functional in these cells. The treatment with the specific pharmacological TLR7 agonist imiquimod demonstrated a significant, dose-dependent inhibition of both adiponectin and resistin protein concentration in cell-culture supernatants (Figure 3a). Of note, the imiquimod dose-dependently reduced adiponectin and resistin mRNA levels in 3T3-L1 adipocytes as well, indicating that the observed effect might be mediated by reduced gene expression levels (Appendix A, Figure A3).

### 2.4. TLR7 Agonist Imiquimod Inhibits Resistin Accumulation in Primary Murine TLR9^wt/wt^ and TLR9^−/−^ Subcutaneous Adipocyte Cell-Culture Supernatants, While Leaving Adiponectin Unchanged in Both TLR9^−/−^ and TLR9^wt/wt^ Adipocyte Cell-Culture Supernatants

Since TLR7 and TLR9 have been suggested as playing interdependent roles in adipose tissue physiology [9,13], we next investigated the impact of the imiquimod treatment in adipocytes isolated from the subcutaneous adipose tissue compartment in TLR9^wt/wt^ versus TLR9^−/−^ mice. The TLR7 agonist imiquimod significantly reduced the resistin accumulation in both the TLR9^wt/wt^ and TLR9^−/−^ adipocyte cell-culture supernatants (*p* = 0.011 for TLR9^wt/wt^ and *p* < 0.001 for TLR9^−/−^ adipocytes, Figure 3b), however, the imiquimod left the adiponectin unchanged in both of the primary murine TLR9^−/−^ and TLR9^wt/wt^ adipocyte cell-culture supernatants (Figure 3c).

### 2.5. TLR7 Agonist Imiquimod Inhibits Glut4 mRNA Expression in 3T3-L1 Adipocytes

We next analyzed Glut1 and Glut4 mRNA expression levels in response to the imiquimod in 3T3-L1 adipocytes in vitro. Remarkably, the imiquimod significantly inhibited the Glut4 mRNA expression in a dose-dependent manner (*p* < 0.001, Figure 3d, right panel). The imiquimod did not significantly impact on Glut1 mRNA expression levels (Figure 3d, left panel).

### 2.6. Cell-Free Nucleic Acids (cfDNA) from 3T3-L1 Adipocytes Treated by TNFα Dose-Dependently Inhibit Adiponectin and Resistin Concentration in Cell-Culture Supernatants

Recently, cell-free nucleic acids (cfDNA) was suggested as acting as a physiological ligand of nucleic-acid sensing pattern recognition receptors (PRR) TLR7 and TLR9 within adipose tissue compartments [9]. To investigate the impact of cfDNA on 3T3-L1 adipocytes, we isolated cfDNA from 3T3-L1 using QIAmp DNA micro kit (Qiagen), as described in the Materials and Methods section. Prior to cfDNA isolation, the adipocytes were stimulated with 50 ng/mL TNFα for 18 h. The analyses indicated that the cfDNA generated by this method contained nucleic acids, of which about 40% were bound in double-stranded DNA (dsDNA) (QIAxpert analysis).

**Figure 4:**
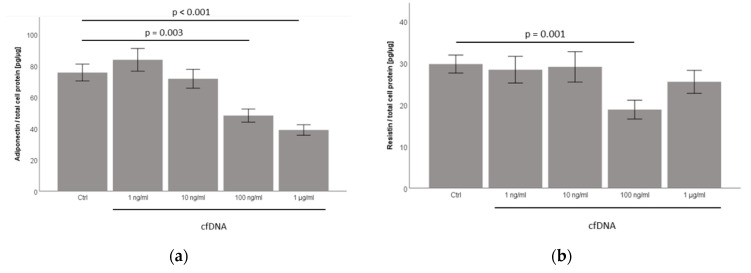

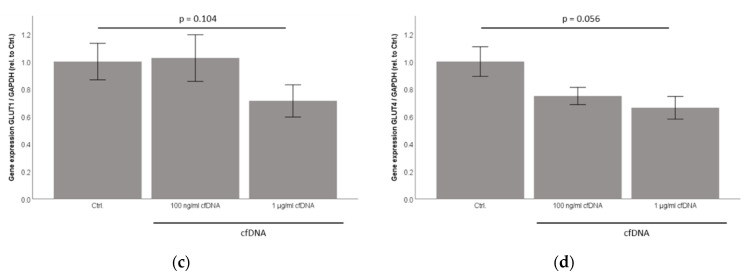
Cell-free nucleic acids (cfDNA) from 3T3-L1 adipocytes stimulated by TNFα dose-dependently inhibit adiponectin and potentially resistin accumulation in cell-culture supernatants and potentially inhibit Glut4 mRNA expression in 3T3-L1 adipocytes. Mature 3T3-L1 were treated with different doses of cfDNA (range: 1 ng/mL–1 µg/mL) generated from TNFα-exposed 3T3-L1 adipocytes as described in the Methods section. Adipokine secretion and gene expression profiles were analyzed by ELISA and RT-PCR, respectively. (**a**,**b**) Supernatant adiponectin concentrations were significantly and dose-dependently decreased by cfDNA, whereas resistin levels were merely reduced by cfDNA treatment at a dose of 100 ng/mL cfDNA (*n* = 11–12); (**c**) cfDNA did not significantly impact on Glut1 mRNA expression levels in 3T3-L1 adipocytes (*n* = 17–18); (**d**) cfDNA seemed to inhibit Glut4 mRNA expression in a dose-dependent manner in 3T3-L1 adipocytes with borderline significance (*p* = 0.056) (*n* = 16–18).

**Figure A4:**
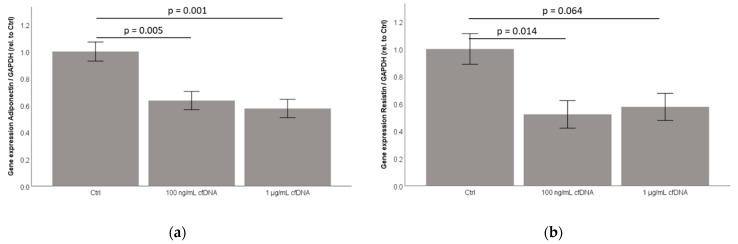
Cell-free nucleic acids (cfDNA) reduce both adiponectin and resistin mRNA levels in 3T3-L1 adipocytes in vitro. Mature 3T3-L1 adipocytes (*n* = 17–18 wells each) were treated with different doses of cfDNA (100 ng/mL, 1 μg/mL) generated from TNF-exposed 3T3-L1 adipocytes as described in the Materials and Methods section for 18 h. After isolation of mRNA applying the RNeasy^®^ Mini Kit (Qiagen, Hilden, Germany), gene expression levels of murine adiponectin and resistin were quantified by reverse transcription and real-time PCR (RT-PCR) of the corresponding cDNA and normalized to GAPDH, as described in the Materials and Methods section. cfDNA reduced both (**a**) adiponectin and (**b**) resistin mRNA levels.

Cell-free nucleic acids (cfDNA) from 3T3-L1 adipocytes significantly inhibited adiponectin concentration in 3T3-L1 adipocyte cell-culture supernatants, while showing an inhibitory effect on resistin concentration at a dose of 100 ng/mL cfDNA (Figure 4a,b). Interestingly, cfDNA dose-dependently reduced adiponectin and resistin mRNA levels in 3T3-L1 adipocytes as well, indicating that the observed effects might be mediated by reduced gene expression levels (Appendix A, Figure A4).

We next analyzed the Glut1 and Glut4 mRNA expression levels in 3T3-L1 adipocytes in response to cfDNA stimulation. Remarkably, cfDNA seemed to inhibit Glut4 mRNA expression in a dose-dependent manner in 3T3-L1 adipocytes with borderline significance (*p* = 0.056, Figure 4d). However, similar to our observation after imiquimod treatment (Figure 3d), cfDNA did not significantly impact on Glut1 mRNA expression (Figure 4c).

### 2.7. Responsiveness of Murine 3T3-L1 Adipocytes to Imiquimod Is Preserved in Co-Culture with Murine J774A.1 Monocytes, However, Co-Cultures Show Significantly Increased Concentrations of MCP1/CCL2 in 3T3-L1 Adipocyte Cell-Culture Supernatants

**Figure 5:**
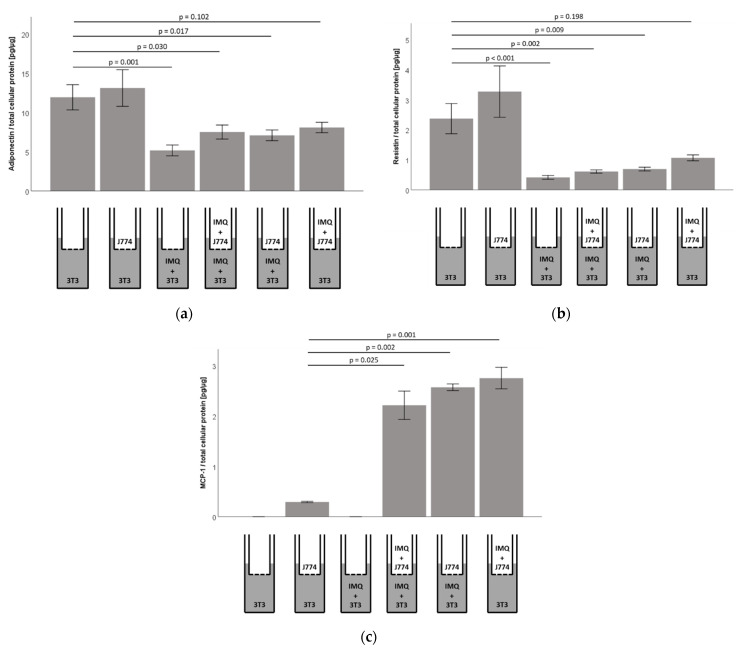
Effects of imiquimod on cell-culture supernatant adipokine concentrations in 3T3-L1 adipocytes are preserved in co-culture with murine J774A.1 monocytes, while co-cultures led to significantly increased levels of MCP1/CCL2 in 3T3-L1 adipocyte cell-culture supernatants. Mature 3T3-L1 adipocytes were treated with IMQ (+IMQ) separately (3T3) and in co-culture (3T3 + J774) with J774A.1 monocytes and adipokine concentrations in cell-culture supernatants in the adipocyte compartment were analyzed by ELISA. (**a**) Treatment of the adipocyte compartment and of both the adipocyte and monocyte compartment led to significantly reduced adiponectin levels in the supernatant of the adipocyte compartment. Inhibition of adiponectin accumulation in cell-culture supernatants was not significant when IMQ treatment was confined to the monocyte compartment (*n* = 5–6); (**b**) treatment of the adipocyte compartment and of both the adipocyte and monocyte compartment led to significantly reduced resistin levels in the supernatant of the adipocyte compartment. Inhibition of resistin accumulation in cell-culture supernatants was not significant when IMQ treatment was confined to the monocyte compartment (*n* = 6); (**c**) treatment of the adipocyte compartment, of both the adipocyte and monocyte compartment and of the monocyte compartment alone led to significantly increased MCP1/CCL2 levels in the supernatant of the adipocyte compartment. We did not detect significant levels of MCP1/CCL2 in supernatants of 3T3-L1 adipocytes without monocyte co-cultures (*n* = 6).

**Figure A5:**
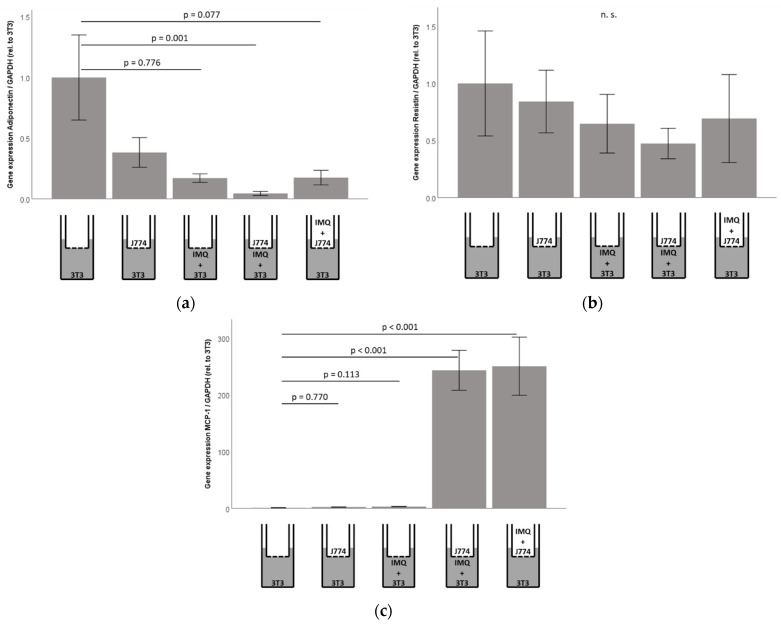
Imiquimod reduces adiponectin while increasing MCP1/CCL2 mRNA levels in 3T3-L1 adipocytes in co-culture with murine J774A.1 monocytes in vitro. Mature 3T3-L1 adipocytes were treated with IMQ (+IMQ) separately (3T3) and in co-culture (3T3 + J774) with J774A.1 monocytes as described in the Methods section for 18 h (*n* = 7–12 wells each). After isolation of mRNA from the adipocytes applying the RNeasy^®^ Mini Kit (Qiagen, Hilden, Germany), gene expression levels of murine adiponectin, resistin and MCP1/CCL2 were quantified by reverse transcription and real-time PCR (RT-PCR) of the corresponding cDNA and normalized to GAPDH, as described in the Materials and Methods section. Remarkably, imiquimod significantly reduced (**a**) adiponectin while increasing (**c**) MCP1/CCL2 mRNA levels in adipocytes in co-culture with monocytes; (**b**) Resistin mRNA levels were not significantly affected. (n.s.—not significant).

To test if the adipocytic TLR7 signaling is modulated by neighboring monocytes, we established co-culture conditions, using murine 3T3-L1 adipocytes (culture dishes) and murine J774A.1 monocytes (inserts), as described in the Materials and Methods section. Co-culture itself did not impact on adiponectin or resistin concentrations in adipocyte cell-culture supernatants (Figure 5a,b). Furthermore, imiquimod-dependent inhibition of resistin and adiponectin concentrations in cell culture supernatants of adipocytes (Figure 3a) was preserved in co-culture with monocytes (Figure 5a,b). The stimulation of the monocyte compartment with imiquimod led to reduced adiponectin and resistin concentrations in the cell-culture supernatants in the adipocyte compartment as well, however, this did not reach statistical significance (Figure 5a,b). Of note, the analyses of adiponectin and resistin mRNA levels in the adipocyte compartment in co-culture with monocytes revealed similarly reduced adiponectin mRNA levels, while the resistin mRNA levels were not significantly altered, indicating that the resistin levels in the supernatants might be influenced by both adipocytes and monocytes (Appendix A, Figure A5).

The MCP1/CCL2 secretion into the cell culture supernatants was not detectable in unstimulated 3T3-L1 adipocytes (Figure 5c). However, we found significant MCP1/CCL2 protein levels in the adipocyte compartment in co-culture with J774A.1 monocytes, which was further and significantly increased after stimulation of the adipocytes and/or monocytes with imiquimod (Figure 5c). Remarkably, similar results were found regarding MCP1/CCL2 mRNA levels in the adipocyte compartment in co-culture with monocytes (Appendix A, Figure A5).

### 2.8. TLR7 mRNA Is Expressed in Similar Levels in Murine Subcutaneous and Intra-Abdominal Adipose Tissue, and TLR7 and TLR9 mRNA Expression in Murine Adipose Tissues Are Strongly Correlated to Each Other

The intracellular PRRs TLR7 and TLR9 have been suggested as participating in the metabolically induced inflammation (metaflammation) in adipose tissue compartments [5,6,7,9], and we recently demonstrated that the TLR9 mRNA is increased in visceral adipose tissue over subcutaneous adipose tissue in human obese patients, but not in mice [13]. The inflammatory changes within the visceral adipose tissue compartment are associated with a metabolic deterioration in patients [7,9,14]. We therefore asked if the TLR7 expression levels differ between murine intra-abdominal and subcutaneous adipose tissue compartments.

**Figure 6:**
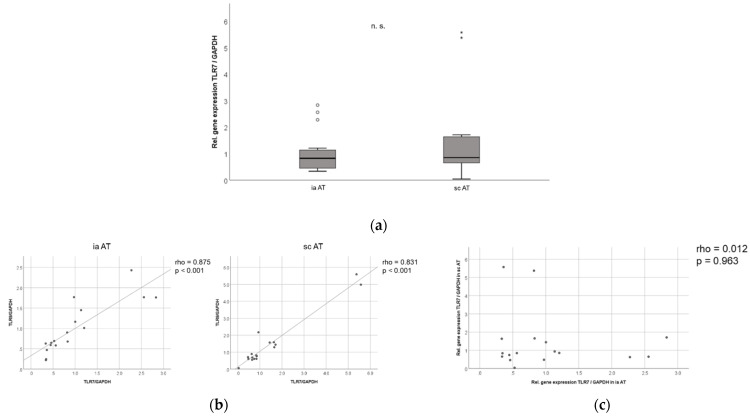
TLR7 is expressed with similar levels in murine subcutaneous and intra-abdominal adipose tissue and strongly correlates with TLR9 mRNA expression. Intra-abdominal (ia) and subcutaneous (sc) adipose tissues (AT) were isolated from female (*n* = 5) and male (*n* = 12) TLR9^wt/wt^ mice, and TLR7 gene expression (normalized to GAPDH expression) was analyzed using RT-PCR. (**a**) No significant differences were observed in TLR7 expression levels between intra-abdominal and subcutaneous AT (*n* = 17); (**b**) there was a strong correlation of TLR7 and TLR9 mRNA levels in both fat compartments (*n* = 17); (**c**) no correlation was observed between intra-abdominal and subcutaneous TLR7 expression levels (*n* = 17). (n.s.—not significant).

Similar to the TLR9 expression levels [13], we did not detect significant differences in the overall TLR7 mRNA expression in the subcutaneous and intra-abdominal adipose tissue compartments in mice (Figure 6a). Similarly, there was no significant difference in the intra-abdominal or subcutaneous TLR7 mRNA expression between male (*n* = 12) and female (*n* = 5) mice. Of note, there was a strong correlation between the TLR7 and TLR9 mRNA expression levels in both the murine intra-abdominal and subcutaneous adipose tissue compartments (*p* < 0.001, rho = 0.875 for intra-abdominal and *p* < 0.001, rho = 0.831 for subcutaneous AT; Figure 6b), indicating that TLR7 and TLR9 might be regulated in parallel in these murine adipose depots. However, we found no correlation in the TLR7 mRNA expression between intra-abdominal and subcutaneous AT (*p* = 0.963, rho = 0.012), hinting at a differential regulation in the murine visceral as opposed to subcutaneous adipose depots (Figure 6c).

### 2.9. TLR7 Is Expressed in Human Adipose Tissue Compartments, and TLR7 Is Significantly Increased in Visceral as Compared to Subcutaneous Adipose Tissue in Non-Diabetic Obese Patients Undergoing Bariatric Surgery

**Figure 7:**
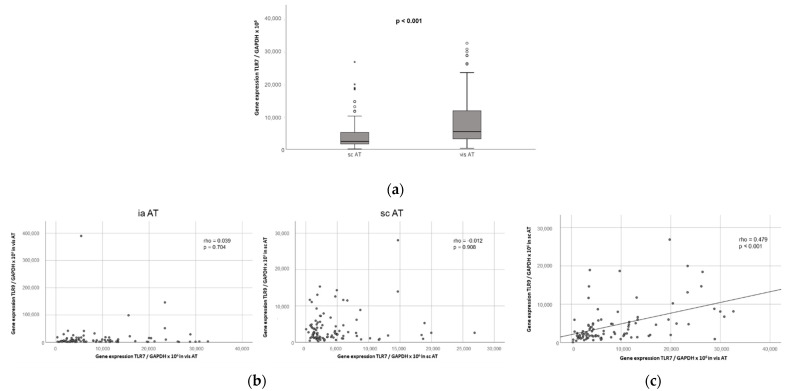

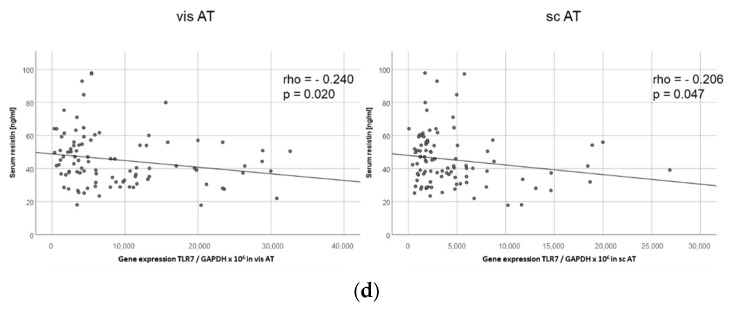
TLR7 is significantly increased in visceral as compared to subcutaneous adipose tissue in non-diabetic obese patients undergoing bariatric surgery and negatively correlates with resistin serum levels. TLR7 gene expression (normalized to GAPDH expression) was quantified via RT-PCR in surgically obtained paired samples of subcutaneous (sc) and visceral (vis) adipose tissue (AT) from 95 obese, non-diabetic patients (14 men, 81 women) undergoing bariatric surgery. (**a**) TLR7 expression levels were significantly increased in visceral as compared to subcutaneous AT (*n* = 95); (**b**) no significant correlation was observed between TLR7 and TLR9 mRNA expression in visceral or subcutaneous AT (*n* = 95); (**c**) visceral AT TLR7 expression was significantly correlated to subcutaneous TLR7 expression (*n* = 95); (**d**) both visceral and subcutaneous AT TLR7 expression levels correlated negatively with resistin levels in the systemic circulation (*n* = 94).

We found that the TLR7 mRNA is expressed in human visceral and subcutaneous adipose tissue compartments in 95 obese, non-diabetic patients (mean age: 37 ± 10 years (range: 19–58 years); mean BMI: 54.3 ± 7.0 kg/m^2^ (range: 40.9–83.7 kg/m^2^)) undergoing bariatric surgery (Figure 7a). Of note, the TLR7 mRNA expression was significantly increased in visceral over subcutaneous adipose tissue in these patients (*p* < 0.001, Figure 7a), indicating that TLR7 might play an important role in this adipose deposit, which is highly relevant for metabolic inflammation and its sequelae [15,16]. These results match our recent findings indicating a significantly increased TLR9 mRNA expression in visceral over subcutaneous adipose tissue in obese patients [13]. There were no significant differences in male versus female TLR7 mRNA expression in the visceral and subcutaneous AT samples in these patients. Interestingly, as opposed to murine samples, we found no correlation between the TLR7 and TLR9 mRNA expression levels in visceral and subcutaneous adipose tissue in human obese patients (Figure 7b), indicating that in human obese patients, TLR7 and TLR9 are regulated independently from each other. However, we found a strong correlation between the TLR7 mRNA expression in visceral and subcutaneous AT specimen (rho = 0.479, *p* < 0.001; Figure 7c), hinting at a possible shared regulation of TLR7 in different human adipose tissue depots. Of note, no significant correlations were detected between TLR7 expression and the clinical and anthropometric parameters that we investigated (age, body weight, body mass index (BMI), waist-to-hip ratio (WHR), fat mass, excessive weight; serum triglycerides, total cholesterol and LDL cholesterol) (Appendix B, Table A1 and Table A2).

**Figure A6:**
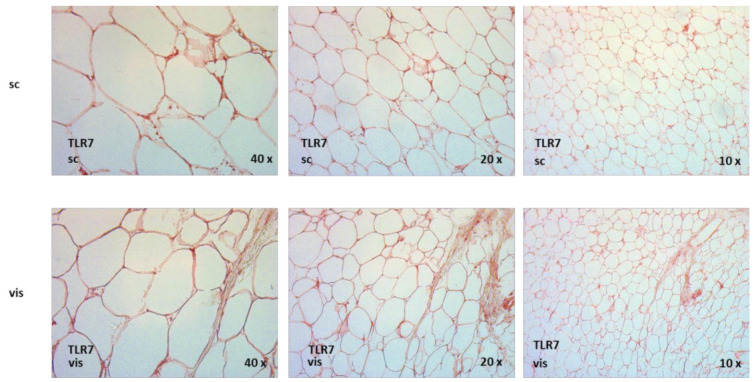
TLR7 protein is expressed in visceral and subcutaneous adipose tissue of obese, non-diabetic patients. TLR7 protein expression was detected and visualized via immunohistochemistry in visceral (vis) and subcutaneous (sc) adipose tissue of obese, non-diabetic patients (representative sections are shown).

The immunohistochemical studies demonstrated that the TLR7 protein is expressed in visceral and in subcutaneous adipose tissues of obese, non-diabetic patients (Appendix A, Figure A6). However, our immunohistochemical analyses did not allow for a quantitative assessment or comparisons of the TLR7 protein expression levels between different patient groups. These analyses should be the focus of future extensive studies. The representative immunohistochemical analyses of the TLR7 protein expression in human visceral and subcutaneous adipose tissue compartments are shown in Appendix A, Figure A6.

### 2.10. TLR7 Expression Negatively Correlates with Resistin Serum Levels in Both Visceral and Subcutaneous Adipose Tissue in Human Obese Patients

Remarkably, when we correlated the TLR7 mRNA expression to systemic adipokine levels in human obese patients, we found that the TLR7 expression in visceral (rho = −0.240, *p* = 0.020) and subcutaneous (rho = −0.206, *p* = 0.047) adipose tissues correlated negatively with the resistin serum levels (Figure 7d). These observations differ from the TLR9 expression levels, which negatively correlated with the resistin serum levels in subcutaneous adipose tissue and positively correlated with the resistin serum levels in the visceral adipose tissue of obese patients [13]. No significant correlations were found between the visceral or subcutaneous TLR7 expression and systemic leptin, adiponectin, C1q/TNF-related protein 3 (CTRP3) or CRP levels, while a borderline significant negative correlation was observed between the TLR7 expression and progranulin serum levels (rho = −0.215, *p* = 0.043 for subcutaneous and rho = −0.202, *p* = 0.057 for visceral adipose tissue TLR7 expression). Table A1 and Table A2 in Appendix B summarize the results of our correlation analyses.

## 3. Discussion

The current study provides a detailed analysis of the TLR7 gene and protein expression in human and murine adipocytes and adipose tissues. In the 3T3-L1 adipocytes, TLR7 is induced earlier during adipocyte development as compared to the endosomal oligonucleotide-sensing TLR9, which shows a gradual increase from day 3 to day 9 [13]. Similar to TLR9 [13], the immunocytochemical analyses indicated a cytosolic localization of TLR7 in adipocytes, matching its endosomal localization in other cell types [8]. Both TLR7 and TLR9 sense nucleic acids and have been postulated to participate in metabolically induced inflammation in the context of obesity and insulin resistance [8,9]. Interestingly, the TLR9 signaling limits the TLR7-mediated immune responses to ssRNA and CpG DNA self-antigens in B-cells in lupus erythematosus patients [17]. Since our group recently demonstrated that the TLR9 signaling has an anti-inflammatory effect in adipocytes [13], TLR9 potentially intersects with the TLR7 signaling in adipocytes in a similar way. Future studies will have to analyze in more detail the molecular basis of the interactions between TLR7 and TLR9 in different cell types and cellular compartments in the context of adipose inflammation.

Resistin is regarded as a key pro-inflammatory adipokine in diabetes mellitus and metabolic syndrome [18]. Remarkably, we found that the specific pharmacological TLR7 agonist imiquimod dose-dependently reduced the concentration of pro-inflammatory/insulin-desensitizing resistin in cell-culture supernatants of 3T3-L1 adipocytes in vitro, indicating the possible anti-inflammatory effects of the TLR7 signaling in mature adipocytes. Imiquimod significantly and dose-dependently reduced resistin concentration in cell-culture supernatants in primary murine adipocytes from both TLR9^wt/wt^- and TLR9^−/−^-mice as well, indicating that these effects are preserved in the absence of TLR9.

The cfDNA levels are increased in the systemic circulation of obese and insulin resistant individuals and were suggested as a possible physiological ligand of nucleic acid-sensing PRRs in adipose tissues [9]. Remarkably, cfDNA significantly inhibited adiponectin secretion in 3T3-L1 adipocytes in vitro, while resistin seemed to be reduced at a dose of 100 ng/mL cfDNA. We assume that not only TLR7, but also other nucleic-acid sensing PRRs, for example TLR3 and TLR8, contribute to the danger-associated molecular pattern (DAMP) sensing of cell-free nucleic acids. Of note, the PRRs can have both beneficial and deleterious effects during metaflammation: for example, a protective role during metabolically induced inflammatory changes has been particularly described for TLR9 [13], NOD2 [19,20] and for TLR5 [21], while inflammasome-mediated caspase-1 activation as well as Nlrp3 signaling in adipocytes renders these cells insulin resistant [22].

Glut4 is the most prominent glucose transporter isoform in adipose tissue, and its inhibition is expected to strongly reduce insulin-dependent adipocytic glucose uptake [23]. Interestingly, the TLR7 agonist imiquimod dose-dependently reduced adipocyte Glut4 mRNA, while cfDNA seemed to inhibit Glut4 expression as well: these results indicate the possible unfavorable metabolic effects of the adipocytic TLR7 signaling. Of note, Glut1 expression is not significantly affected by imiquimod or cfDNA, however, constitutionally expressed adipocytic Glut1 plays a negligible role for insulin-stimulated adipocytic glucose transport [24].

Physiologically, a strong and close interaction between adipocytes and monocytes within adipose tissue compartments has been described [25,26,27]: adipocytes release pro-inflammatory adipokines, for example MCP1/CCL2, TNFα and resistin as well as saturated free fatty acids, which activate adipose tissue-resident macrophages into a pro-inflammatory M1 phenotype, and additionally recruit monocytes from the circulation. The activated macrophages release pro-inflammatory mediators, for example IL-1, IL-6 and TNFα, which act on the adipocytes in a paracrine manner, resulting in increased pro-inflammatory adipokine secretion and the suppression of anti-inflammatory adiponectin secretion [28]. On the other hand, the “adipose tissue expandability hypothesis” has recently been proposed: the stroma-vascular cell fraction of the adipose tissue depots contains adipose tissue-derived stem-cells (ASCs), which can lead to the expansion of metabolically favorable (subcutaneous) or unfavorable (visceral) adipose tissue in a genetically predetermined, compartment-specific manner [15]. Multiple molecular pathways regulating the depot-specific ASC recruitment and differentiation have been described (e.g., Wnt, BMP, sirtuins, microRNAs) [15]. Interestingly, the long, noncoding RNA GAS5, in exosomes derived from human ASCs, regulates the TLR7 expression in human dermal fibroblasts [29]. Since we found an increased expression of TLR7 during adipocyte differentiation, these observations are compatible with a potential role for TLR7 during ASC recruitment and differentiation into mature adipocytes. Future studies, including adipocyte differentiation under conditions of TLR7 insufficiency, will have to characterize these interactions in more detail.

Since the TLR7 expression was expectedly much stronger in the stroma-vascular cell (SVC) fraction of murine adipose tissues as compared to the adipocyte fraction, we asked how the macrophages might modulate the TLR7-dependent adipocytic adipokine secretion. Of note, the TLR7-mediated inhibition of resistin and adiponectin concentration in adipocyte cell culture supernatants was preserved in co-culture with monocytes. Interestingly, the stimulation of the monocyte compartment with imiquimod led to a reduced adiponectin and resistin secretion in the adipocyte compartment, however, this did not reach statistical significance. Remarkably, the stimulation of TLR7 with imiquimod resulted in a significantly increased MCP1/CCL2 protein secretion in adipocytes in co-culture with monocytes, which was not observed in adipocytes alone. Since MCP1/CCL2 is a key chemokine, promoting macrophage infiltration into fat tissues and thus metabolic inflammation [30], these data indicate a possible physiological role of the adipocytic TLR7 signaling in the adipocyte–monocyte interaction within adipose tissues.

In human adipose tissue from non-diabetic obese patients, we observed a significant negative correlation between the TLR7 expression in human visceral as well as subcutaneous adipose tissue and systemic resistin serum levels, matching the observed inhibitory effect of the TLR7 signaling on resistin secretion in adipocytes in vitro. Remarkably, we observed significantly higher expression levels in visceral as opposed to subcutaneous adipose tissue, which is mirrored by a similar expression pattern for TLR9 in human adipose tissues in our previous study [13]. Of note, we found a highly significant and strong correlation between the TLR7 and TLR9 gene expression, both in intra-abdominal as well as in subcutaneous adipose tissue compartments in normal-weight mice, indicating a possible interaction between the murine TLR7 and TLR9 gene expression in these compartments. However, we did not find any correlation between the TLR7 and TLR9 expression levels in human subcutaneous nor in visceral adipose tissue. Thus, in human obese patients, TLR7 and TLR9 seem to be regulated independently from each other.

In summary, we demonstrate that the adipocyte-derived cfDNA and TLR7 agonistic imiquimod affect adipokine expression, adipokine concentration in cell-culture supernatants and glucose transporter expression in adipocytes. Presumably, additional TLRs and/or PRRs contribute to the effects observed with cfDNA treatment as opposed to the isolated stimulation of TLR7 by the specific TLR7 ligand, imiquimod. Future studies, including dynamic glucose metabolism analyses, will have to dissect a possible impact of TLR7 and cfDNA on adipocyte cellular metabolism in more detail. It is a limitation that most of the data in our manuscript were generated in murine adipocytes, as well as ex vivo and in vitro cell culture systems, where the TLR9^−/−^-animals/cells could be used. Therefore, future studies will have to confirm the selected results in human adipocytes in vivo and/or ex vivo. For example, an increased TLR7 expression is expected during adipogenesis in human adipose-derived stem cells from both subcutaneous and visceral depots, similar to our observation in 3T3-L1 adipocytes in vitro. These studies will be the focus of future experiments. While we found that the specific pharmacological TLR7 ligand, imiquimod, as well as the proposed ligand cfDNA, impact on the adipocyte gene expression and adipokine secretion, definite proof of the dependency of the observed effects by imiquimod/cfDNA requires experiments using the TLR7 knock-out cells/animals. Furthermore, the impact of the additional TLR7 agonists, for example microRNA specific for TLR7 [31], should be investigated, as well as the effects of co-stimulation of other TLRs, for example, TLR4 by lipopolysaccharide (LPS) treatment, to determine the reciprocal effects of induced inflammation on TLR7-mediated outcomes. These extensive studies will be the focus of future experiments.

## 4. Materials and Methods

### 4.1. Adipocyte Cell Culture and Stimulation Experiments

The murine 3T3-L1-pre-adipocytes [32], as well as the primary murine adipocytes (pmAd) isolated from C57BL/6J and C57BL/6J-Tlr9^M7Btlr^/Mmjax, were cultured at 37 °C and 5% CO_2_ in DMEM (Dulbecco’s Modified Eagle Medium, Biochrom AG, Berlin, Germany) that was supplemented with 10% newborn calf serum (NCS; from Sigma-Aldrich, Deisenhofen, Germany) and 1% penicillin/streptomycin (PAN, Aidenbach, Germany). The cells were differentiated into mature adipocytes at confluence by DMEM/F12/glutamate-medium (Lonza, Basel, Switzerland) supplemented with 20 µM 3-isobutyl-methyl-xanthine (Serva, Heidelberg, Germany), 1 µM corticosterone, 100 nM insulin, 200 µM ascorbate, 2 µg/mL apo-transferrin, 5% fetal calf serum, 1 µM biotin, 17 µM pantothenate, 1% penicillin/streptomycin (all from Sigma Aldrich, Deisenhofen, Germany) and 300 µg/mL Pedersen-fetuin (MP Biomedicals, Illkirch, France) [33,34], using a slightly modified protocol as reported in the literature [32,35,36,37,38]. The formation of the spherical adipocyte phenotype with extensive lipid droplet accumulation was monitored by light-microscopy. The mature adipocytes were used for stimulation experiments, following overnight incubation under serum-free culture conditions. The TLR7 agonist imiquimod (IMQ) was purchased from InvivoGen (San Diego, CA, USA) and dissolved in H_2_O, according to the manufacturer’s recommendations. Different doses of IMQ dissolved in DMEM/F12/glutamate-medium were used for overnight (18 h) stimulation experiments.

To investigate the impact of the cfDNA on the 3T3-L1 adipocytes, we isolated cfDNA from the mature 3T3-L1 adipocytes. Prior to the cfDNA preparation, the adipocytes were exposed to inflammatory conditions for 18 h (37 °C, 5% CO_2_). The inflammatory stress was induced by stimulation with 50 ng/mL tumor necrosis factor α (TNFα) (Biomol, Hamburg, Germany). The cells were then washed with phosphate-buffered saline (PBS) in order to remove the remaining TNFα and were transferred from culture plates into lysis buffer (Qiagen, Hilden, Germany) with cell scrapers. The cfDNA was subsequently isolated from the cell lysates applying QIAamp^®^ DNA Micro Kit, according to the manufacturer’s instructions (Qiagen, Hilden, Germany). The analyses indicated that the cfDNA generated by this method contained nucleic acids, of which about 40% were bound in double-stranded DNA (dsDNA) (QIAxpert analysis). Different doses of the cfDNA were used for overnight (18 h) stimulation experiments, as indicated in the figure legends, while stimulation with solvent control containing no cfDNA served as control for the cfDNA experiments.

The LDH (lactate dehydrogenase) concentration was measured in the supernatants (Cytotoxicity Detection Kit, Roche, Mannheim, Germany) in order to exclude any potential cytotoxic effects of IMQ or cfDNA.

### 4.2. Co-Culture of Adipocytes and Monocytes

The murine J774A.1 monocytes (from American Type Culture Collection, Manassas, VA, USA) were cultured in DMEM/F12/glutamate-medium (Lonza, Basel, Switzerland) supplemented with 10% fetal calf serum. For the co-culture experiments, cells were grown in cell culture dish inserts (from Greiner Bio-One, Frickenhausen, Germany) at a density of 50,000 cells per well and incubated with DMEM/F12/glutamate-medium + 10% FCS for 18 h. After a change to serum-free DMEM/F12/glutamate-medium, inserts with monocytes were placed in culture dishes containing differentiated, mature 3T3-L1 adipocytes. After at least 3 h of co-incubation, adipocytes and monocytes were treated in the respective compartment with either IMQ or solvent control H_2_O, as described above. The cell viability was tested by LDH measurement, in order to exclude the cytotoxic effects of stimulation doses.

After stimulation, the cell culture supernatants were harvested from the adipocyte compartment and adiponectin, resistin and MCP1/CCL2 protein concentrations were measured by ELISA, as described below. Adiponectin, resistin and MCP1/CCL2 protein concentrations were normalized to total protein content of the adipocyte fraction, as described below.

### 4.3. Isolation of Adipocytes and SVCs from Mice

Small portions of the intra-abdominal and subcutaneous adipose tissue from mice were digested with 0.225 U/mL collagenase NB 6 (#17458, SERVA Electrophoresis; Heidelberg, Germany) for 30–60 min at 37 °C. The adipocytes were separated from the stromal vascular cells (SVC) via centrifugation (200 rcf, 10 min, 4 °C) and later transferred into TRIzol^®^-Reagent (Life Technologies GmbH, Darmstadt, Germany) for mRNA isolation, as described below. The purity of the adipocytes versus SVC after our isolation procedure was assessed by real-time PCR, using adiponectin for the adipocyte fraction, and using CD45 (indicating leukocyte lineage) for the SVC fraction of cells, as described in the Results section. The adiponectin was almost exclusively expressed in the adipocyte fraction of cells, indicating that we reached a high purity of adipocytes during our isolation procedure. The CD45, on the other hand, was almost exclusively expressed in the SVC fraction of cells, indicating that—as expected—this fraction contained a significant number of immune cells, which were absent in the adipocyte fraction of cells.

### 4.4. Preparation of Human and Murine Tissues for mRNA Isolation

The cell dissociation from frozen murine intra-abdominal and subcutaneous adipose tissue and from human visceral and subcutaneous adipose tissue was performed using TRIzol^®^-Reagent (Life Technologies GmbH, Darmstadt, Germany) in combination with gentleMACS dissociator and M-tubes (Miltenyi Biotec GmbH, Bergisch Gladbach, Germany).

### 4.5. Quantification of mRNA Expression in Murine 3T3-L1 Adipocytes In Vitro and Ex Vivo in Murine and Human Adipose Tissues

After isolation of mRNA applying the RNeasy^®^ Mini Kit (Qiagen, Hilden, Germany), the gene expression levels of murine TLR7, TLR9, adiponectin, resistin, CD45, glucose transporter 1 (GLUT1) and glucose transporter 4 (GLUT4) and of human TLR7 and TLR9 were quantified by reverse transcription and real-time PCR (RT-PCR) of the corresponding cDNA. The following primer-pairs were used:
Murine adiponectin: 5′-AGGGAGAGAAAGGAGATGCAG-3′/5′-CAGACTTGGGCTCCCACCTC-3′Murine GLUT1: 5′-AGCAGAGGCTTGCTTGTAGA-3′/5′-AACTCCTCAATAACCTTCTGGGG-3′Murine GLUT4: 5′-TGGTTCATTGTGGCAGAGC-3′/5′-CGTAAGGACCCATAGCATCC-3′Murine resistin: 5′-TGCTAAGTCCTCTGCCACGTA-3′/5′-TCAACTGACCGACATCAG GA-3′Murine TLR7: 5′-GGCATTCCCACTAACACCAC-3′/5′-TTGGACCCCAGTAGAACAGG-3′Murine TLR9: 5′-CATCTCCCAACATGGTTCTCC-3′/5′-GCAGAGAAACGGGGTACAGA-3′Murine CD45: 5′-TGACCATGGGTTTGTGGCTC-3′/5′-TCGTTGTGGTAGCATCACTGG-3′Human TLR7: 5′-TCAAGAAAGTTGATGCTATTGGGC-3′/5′-CTGTGCAGTCCACGATCACA-3′Human TLR9: 5′-CCCCCAGCATGGGTTTCT-3′/5′-TGGAGCTCACAGGGTAGGAA-3′

The expression levels of these genes were normalized to gene expression of glyceraldehyde-3-phosphate dehydrogenase (GAPDH) by using primer-pairs 5′ GAGTCCACTGGCGTCTTCAC-3′/5′-CCAGGGGTGCTAAGCAGTT-3′ (human) and 5′-TGTCCGTCGTGGATCTGAC-3′/5′-AGGGAGATGCTCAGTGTTGG-3′ (mouse). Every biological replicate was investigated in two–three technical replicates. According to the MIQE guidelines, technical replicates with a standard deviation of more than 0.3 in quantification cycle number were excluded from the analysis. All of the oligonucleotides used were purchased from Metabion (Martinsried, Germany).

### 4.6. Quantification of Adiponectin and Resistin Protein Concentrations in 3T3-L1 and pmAd Cell Culture Supernatants

The murine adiponectin, resistin and MCP1/CCL2 protein concentrations were measured in duplicates by ELISA techniques (murine adiponectin and resistin DuoSet^®^ ELISA development systems from R&D Systems, Wiesbaden, Germany; murine MCP1/CCL2 ELISA Kit from BioLegend, San Diego, CA, USA) and are expressed as means ± standard error of the mean (SEM). The detection ranges of the applied ELISA kits were 31.2–2000 pg/mL (for adiponectin), 15.6–1000 pg/mL (for murine resistin) and 62.5–4000 pg/mL (for murine MCP-1). The coefficients of variation (CV) of values within duplicate measurements were less than 20%. For 3T3-L1, as well as pmAd cell culture supernatants, the adiponectin and resistin levels were normalized to total cellular protein content in each well.

### 4.7. Immunocytochemistry (ICC)

The 3T3-L1 fibroblasts were differentiated into mature adipocytes, as described above, and shock-frozen by ice-cold acetone (ROTH, Karlsruhe, Germany) for immunocytochemistry. The air-dried cells were incubated in PBS for rehydration. The endogenous peroxidase activity was blocked with 3% H_2_O_2_ (ROTH, Karlsruhe, Germany). To avoid non-specific protein binding, the cells were incubated in 10% bovine serum albumin (BSA, ROTH, Karlsruhe, Germany), 10% fetal calf serum (FCS, Sigma-Aldrich, Steinheim, Germany) and 10% chicken serum (Sigma-Aldrich, Steinheim, Germany), followed by 3 h incubation in a moist chamber with a polyclonal anti-TLR7 antibody from rabbit (2 µg/mL in 1% BSA; Invitrogen, Carlsbad, CA, USA). The cells were then stained with peroxidase-conjugated goat anti-rabbit IgG (1.6 µg/mL in 1% BSA; Jackson Immuno Research, West Grove, Pennsylvania, USA) for 90 min. The color development with 3-amino-9-ethylcarbazole (AEC) substrate (Vector Laboratories, Burlingame, CA, USA) at room temperature was stopped after microscopic examination. The rabbit isotype-matched IgG sera (ab37415; Abcam, Cambridge, UK) served as an isotype control. Parallel experiments without primary antibodies were carried out as negative controls.

### 4.8. Immunohistochemistry (IHC)

The murine and human adipose tissue samples were fixed in ROTI^®^Histofix 4% (Carl Roth, Karlsruhe, Germany) for 24 h and later embedded in paraffin. The paraffin was removed with xylene (ROTH, Karlsruhe, Germany) and by consecutive washing steps with 100%, 96% and 70% ethanol (ROTH, Karlsruhe, Germany). The endogenous peroxidase activity was blocked with 3% H_2_O_2_. Tissue samples were incubated for 60 min in citrate buffer at 60 °C. Non-specific binding sites were blocked with 5% BSA for 60 min in a humid chamber followed by an overnight incubation in a moist chamber with a polyclonal anti-TLR7 antibody from rabbit (2 µg/mL in 1% BSA; Invitrogen, Carlsbad California, USA). The cells were then stained with peroxidase-conjugated goat anti-rabbit IgG (1.6 µg/mL in 1% BSA; Jackson Immuno Research, West Grove, Pennsylvania, USA) for 90 min. The color development with 3-amino-9-ethylcarbazole (AEC) substrate (Vector Laboratories, Burlingame, California) at room temperature was stopped after microscopic examination. The rabbit isotype-matched IgG sera (ab37415; Abcam, Cambridge, UK) served as an isotype control. Parallel experiments without primary antibodies were carried out as negative controls.

### 4.9. Animals

Healthy C57BL/6J and C57BL/6J-Tlr9^M7Btlr^/Mmjax mice were bred under standard conditions and were fed a chow diet ad libitum until euthanasia by carbon dioxide asphyxiation for organ and tissue resection. The intra-abdominal and subcutaneous adipose tissue was harvested from male mice (18–28 weeks old, 24–38 g) for immunohistochemical analysis and for immediate fractionation of cells. Additional intra-abdominal and subcutaneous adipose tissue was harvested from either male (31–35 weeks old, 29–39 g) or female mice (16–24 weeks old, 20–27 g; gender is indicated in the respective figure legend) for isolation of primary murine preadipocytes. The animal experiments were performed at the University of Giessen, Germany, and all of the animal studies were approved by the local government agency.

### 4.10. Patients and Study Cohort

Paired human visceral and subcutaneous adipose tissue samples were obtained from 95 obese, non-diabetic patients (81 females, 14 males; mean age: 37 ± 10 years (range: 19–58 years); mean BMI: 54.3 ± 7.0 kg/m^2^ (range: 40.9–83.7 kg/m^2^)) undergoing bariatric surgery and were analyzed for TLR7 mRNA expression by RT-PCR, as described above. Additionally, the serum samples were obtained from these patients for systemic adipokine level analysis by ELISA, as described above. The clinical and experimental work on this study cohort was completed by the ROBS (Research in Obesity and Bariatric Surgery) study group at the Giessen University Hospital, Germany (see Acknowledgements) on the basis of written informed patient consent and approval of the local Ethical Committee (AZ 101/14).

### 4.11. Statistical Analysis

Unless indicated otherwise, the data are given as mean values ± standard error of the mean (SEM). For calculating the mean values ± SEM and standard deviation (SD), a statistical software package (SPSS 26.0; Armonk, NY, USA) was used. The values with a higher discrepancy from the mean than twice the amount of the standard deviation were excluded from analysis. The mean values were compared by the non-parametric Mann–Whitney U-test for two independent samples and by Kruskal–Wallis-H-test for k independent samples. The correlation analysis was completed with the Spearman-rho test for linear variables. A *p*-value below 0.05 (two-tailed) was considered as statistically significant.

## 5. Conclusions

Our data show that adipocytes express the functional TLR7. The immunohistochemical analyses indicate a cytosolic localization, and TLR7 seems to have anti-inflammatory effects on adipocytes by reducing resistin secretion. The cfDNA generated from cultured adipocytes impacts on adipocyte adipokine levels in cell-culture supernatants and on the expression of genes relevant to adipocyte physiology. Future studies should focus on the impact of TLR7 and cfDNA on adipocyte cellular metabolism and physiology.

## Data Availability

The data presented in this study are available on reasonable request from the corresponding author.

## Appendix B

**Table A1:** Correlation analysis of TLR7 mRNA expression in subcutaneous adipose tissue with clinical and anthropometric parameters and with systemic adipokine levels. *n* = 95 obese, non-diabetic patients (14 men, 81 women). Correlation analysis was done by using the Spearman-rho test, and the respective Spearman rho and *p*-values are given. The bold in the table marks statistically significant correlations.

	rho	*p*
**TLR7 mRNA (visceral AT)**	**+0.479**	**<0.001**
Age	−0.024	0.818
Body weight	−0.015	0.888
BMI	−0.024	0.820
Body fat percentage	−0.110	0.322
WHR	+0.030	0.796
Excessive weight	−0.026	0.804
Total cholesterol	−0.069	0.528
LDL cholesterol	+0.078	0.477
**HDL cholesterol**	**−0.226**	**0.038**
Triglycerides	+0.107	0.328
CRP	+0.026	0.799
Adiponectin	−0.159	0.125
Leptin	−0.140	0.179
**Resistin**	**−0.206**	**0.047**
CTRP3	−0.166	0.112
**Progranulin**	**−0.215**	**0.043**

**Table A2:** Correlation analysis of TLR7 mRNA expression in visceral adipose tissue with clinical and anthropometric parameters and with systemic adipokine levels. *n* = 95 obese, non-diabetic patients (14 men, 81 women). Correlation analysis was done by using the Spearman-rho test, and the respective Spearman rho and *p*-values are given. The bold in the table marks statistically significant correlations.

	rho	*p*
**TLR7 mRNA (subcutaneous AT)**	**+0.479**	**<0.001**
Age	−0.101	0.331
Body weight	−0.103	0.320
BMI	−0.038	0.714
Body fat percentage	−0.142	0.200
WHR	+0.016	0.886
Excessive weight	−0.091	0.382
Total cholesterol	−0.027	0.808
LDL cholesterol	+0.045	0.680
HDL cholesterol	−0.016	0.884
Triglycerides	−0.001	0.996
CRP	+0.187	0.070
Adiponectin	−0.049	0.636
Leptin	−0.107	0.305
**Resistin**	**−0.240**	**0.020**
CTRP3	−0.185	0.075
Progranulin	−0.202	0.057

## References

[1] Hotamisligil G.S. (2017). Inflammation, metaflammation and immunometabolic disorders. Nature.

[2] Wang X., Wang Y., Antony V., Sun H., Liang G. (2020). Metabolism-Associated Molecular Patterns (MAMPs). Trends Endocrinol. Metab..

[3] Schäffler A., Schölmerich J. (2010). Innate immunity and adipose tissue biology. Trends Immunol..

[4] Kim S.J., Choi Y., Choi Y.H., Park T. (2012). Obesity Activates Toll-Like Receptor-Mediated Proinflammatory Signaling Cascades in the Adipose Tissue of Mice. J. Nutr. Biochem..

[5] Hong C.-P., Yun C.H., Lee G.-W., Park A., Kim Y.-M., Jang M.H. (2015). TLR9 regulates adipose tissue inflammation and obesity-related metabolic disorders. Obesity.

[6] Nishimoto S., Fukuda D., Higashikuni Y., Tanaka K., Hirata Y., Murata C., Kim-Kaneyama J.-R., Sato F., Bando M., Yagi S. (2016). Obesity-induced DNA released from adipocytes stimulates chronic adipose tissue inflammation and insulin resistance. Sci. Adv..

[7] Ghosh A.R., Bhattacharya R., Bhattacharya S., Nargis T., Rahaman O., Duttagupta P., Raychaudhuri D., Liu C.S.C., Roy S., Ghosh P. (2016). Adipose Recruitment and Activation of Plasmacytoid Dendritic Cells Fuel Metaflammation. Diabetes.

[8] O’Neill L.A.J., Golenbock D., Bowie A.G. (2013). The history of Toll-like receptors—Redefining innate immunity. Nat. Rev. Immunol..

[9] Revelo X.S., Ghazarian M., Chng M.H.Y., Luck H., Kim J.H., Zeng K., Shi S.Y., Tsai S., Lei H., Kenkel J. (2016). Nucleic Acid-Targeting Pathways Promote Inflammation in Obesity-Related Insulin Resistance. Cell Rep..

[10] Latorre J., Moreno-Navarrete J.M., Sabater M., Buxo M., Rodriguez-Hermosa J.I., Girones J., Fort J.M., Vilallonga R., Ricart W., Simo R. (2018). Decreased TLR3 in Hyperplastic Adipose Tissue, Blood and Inflamed Adipocytes is Related to Metabolic Inflammation. Cell. Physiol. Biochem..

[11] Maeda K., Akira S. (2016). TLR7 Structure: Cut in Z-Loop. Immunity.

[12] Zhang Z., Ohto U., Shibata T., Krayukhina E., Taoka M., Yamauchi Y., Tanji H., Isobe T., Uchiyama S., Miyake K. (2016). Structural Analysis Reveals that Toll-like Receptor 7 Is a Dual Receptor for Guanosine and Single-Stranded RNA. Immunity.

[13] Thomalla M., Schmid A., Neumann E., Pfefferle P.I., Muller-Ladner U., Schaffler A., Karrasch T. (2019). Evidence of an Anti-Inflammatory Toll-Like Receptor 9 (Tlr 9) Pathway in Adipocytes. J. Endocrinol..

[14] Hardy O.T., Czech M.P., Corvera S. (2012). What Causes the Insulin Resistance Underlying Obesity?. Curr. Opin. Endocrinol. Diabetes Obes..

[15] Porro S., Genchi V.A., Cignarelli A., Natalicchio A., Laviola L., Giorgino F., Perrini S. (2021). Dysmetabolic Adipose Tissue in Obesity: Morphological and Functional Characteristics of Adipose Stem Cells and Mature Adipocytes in Healthy and Unhealthy Obese Subjects. J. Endocrinol. Investig..

[16] Cariou B. (2022). The Metabolic Triad of Non-Alcoholic Fatty Liver Disease, Visceral Adiposity and Type 2 Diabetes: Implications for Treatment. Diabetes Obes. Metab..

[17] Fillatreau S., Manfroi B., Dörner T. (2020). Toll-like receptor signalling in B cells during systemic lupus erythematosus. Nat. Rev. Rheumatol..

[18] Abate N., Sallam H.S., Rizzo M., Nikolic D., Obradovic M., Bjelogrlic P., Isenovic E.R. (2014). Resistin: An Inflammatory Cytokine. Role in Cardiovascular Diseases, Diabetes and the Metabolic Syndrome. Curr. Pharm. Des..

[19] Denou E., Lolmede K., Garidou L., Pomie C., Chabo C., Lau T.C., Fullerton M.D., Nigro G., Zakaroff-Girard A., Luche E. (2015). Defective Nod2 Peptidoglycan Sensing Promotes Diet-Induced Inflammation, Dysbiosis, and Insulin Resistance. EMBO Mol. Med..

[20] Cavallari J.F., Fullerton M.D., Duggan B.M., Foley K.P., Denou E., Smith B.K., Desjardins E.M., Henriksbo B.D., Kim K.J., Tuinema B.R. (2017). Muramyl Dipeptide-Based Postbiotics Mitigate Obesity-Induced Insulin Resistance via IRF4. Cell Metab..

[21] Vijay-Kumar M., Aitken J.D., Carvalho F.A., Cullender T.C., Mwangi S., Srinivasan S., Sitaraman S.V., Knight R., Ley R.E., Gewirtz A.T. (2010). Metabolic Syndrome and Altered Gut Microbiota in Mice Lacking Toll-Like Receptor 5. Science.

[22] Stienstra R., Joosten L.A., Koenen T., van Tits B., van Diepen J.A., van den Berg S.A., Rensen P.C.N., Voshol P.J., Fantuzzi G., Hijmans A. (2010). The Inflammasome-Mediated Caspase-1 Activation Controls Adipocyte Differentiation and Insulin Sensitivity. Cell Metab..

[23] Govers R. (2014). Molecular mechanisms of GLUT4 regulation in adipocytes. Diabetes Metab..

[24] Chadt A., Al-Hasani H. (2020). Glucose transporters in adipose tissue, liver, and skeletal muscle in metabolic health and disease. Pflugers Arch..

[25] Engin A.B. (2017). Adipocyte-Macrophage Cross-Talk in Obesity. Obesity Lipotoxicity.

[26] Bai Y., Sun Q. (2015). Macrophage recruitment in obese adipose tissue. Obes. Rev..

[27] Ouchi N., Parker J.L., Lugus J.J., Walsh K. (2011). Adipokines in inflammation and metabolic disease. Nat. Rev. Immunol..

[28] Suganami T., Nishida J., Ogawa Y. (2005). A Paracrine Loop between Adipocytes and Macrophages Aggravates Inflammatory Changes: Role of Free Fatty Acids and Tumor Necrosis Factor Alpha. Arterioscler. Thromb. Vasc. Biol..

[29] Patel R.S., Impreso S., Lui A., Vidyarthi G., Albear P., Patel N.A. (2022). Long Noncoding Rna Gas5 Contained in Exosomes Derived from Human Adipose Stem Cells Promotes Repair and Modulates Inflammation in a Chronic Dermal Wound Healing Model. Biology.

[30] Kanda H., Tateya S., Tamori Y., Kotani K., Hiasa K., Kitazawa R., Kitazawa S., Miyachi H., Maeda S., Egashira K. (2006). Mcp-1 Contributes to Macrophage Infiltration into Adipose Tissue, Insulin Resistance, and Hepatic Steatosis in Obesity. J. Clin. Investig..

[31] Wu N., Morsey B.M., Emanuel K.M., Fox H.S. (2021). Sequence-specific extracellular microRNAs activate TLR7 and induce cytokine secretion and leukocyte migration. Mol. Cell. Biochem..

[32] Green H., Kehinde O. (1975). An Established Preadipose Cell Line and Its Differentiation in Culture. Ii. Factors Affecting the Adipose Conversion. Cell.

[33] Zaitsu H., Serrero G. (1990). Pedersen fetuin contains three adipogenic factors with distinct biochemical characteristics. J. Cell. Physiol..

[34] Bachmeier M., Löffler G. (1994). Adipogenic Activities in Commercial Preparations of Fetuin. Horm. Metab. Res..

[35] MacDougald O.A., Lane M.D. (1995). Transcriptional Regulation of Gene Expression During Adipocyte Differentiation. Annu. Rev. Biochem..

[36] Green H., Kehinde O. (1979). Formation of normally differentiated subcutaneous fat pads by an established preadipose cell line. J. Cell. Physiol..

[37] Green H., Meuth M. (1974). An established pre-adipose cell line and its differentiation in culture. Cell.

[38] Cornelius P., MacDougald O.A., Lane M.D. (1994). Regulation of Adipocyte Development. Annu. Rev. Nutr..

[39] Hochberg A., Patz M., Karrasch T., Schäffler A., Schmid A. (2021). Serum Levels and Adipose Tissue Gene Expression of Cathelicidin Antimicrobial Peptide (CAMP) in Obesity and During Weight Loss. Horm. Metab. Res..

[40] Brock J., Schmid A., Karrasch T., Pfefferle P., Schlegel J., Busse I., Hauenschild A., Schmidt B., Koukou M., Arapogianni E. (2019). Progranulin serum levels and gene expression in subcutaneous vs visceral adipose tissue of severely obese patients undergoing bariatric surgery. Clin. Endocrinol..

[41] Schmid A., Gehl J., Thomalla M., Hochberg A., Kreiß A., Patz M., Karrasch T., Schäffler A. (2020). Downregulation of CTRP-3 by Weight Loss In Vivo and by Bile Acids and Incretins in Adipocytes In Vitro. Int. J. Mol. Sci..

